# Effectiveness of mixture of aspirin and ethanol regimen in the management of geographic tongue: a prospective study

**DOI:** 10.1097/MS9.0000000000000785

**Published:** 2023-05-10

**Authors:** Karpal S. Sohal, Sira S. Owibingire, Jeremiah R. Moshy, Walter A. Odhiambo, Boniphace Lubasha

**Affiliations:** a Department of Oral and Maxillofacial Surgery, Muhimbili University of Health and Allied Sciences; b School of Dentistry, Muhimbili University of Health and Allied Sciences, Dar es Salaam, Tanzania; c Department of Oral and Maxillofacial Surgery, University of Nairobi, Nairobi, Kenya

**Keywords:** aspirin, ethanol, geographic tongue, symptomatic

## Abstract

Geographic tongue is usually an asymptomatic inflammatory disorder of the tongue; however, symptomatic cases do occur and require treatment. Several management strategies have been reported yet they are neither specific nor curative.

**Objective:**

The objective was to evaluate the effectiveness of an aspirin-ethanol mixture in the management of geographic tongue.

**Methods:**

In this prospective study, an analysis of medical reports of symptomatic cases of geographic tongue managed using an aspirin-ethanol mixture was done. The treatment regimen involved dissolving 3 mg of aspirin (acetylsalicylic acid) into 1 ml of 70% v/v ethanol. Then a sterile gauze is soaked in the prepared mixture and is topically used to clean the tongue with gentle pressure for 2–3 min.

**Results:**

The records of 23 patients were analyzed. There were more females (19, 82.6%), and the age range of patients was 15–43 years (mean age of 23.4 years). The symptoms that the patient described included: oral discomfort, burning sensations of the tongue, pain, and loss of taste. Only 21 (91.3%) patients were asymptomatic at 3 months of follow-up.

**Conclusions:**

Short-term topical application of the aspirin-ethanol mixture was shown to be effective in treating symptomatic geographic tongue.

## Introduction

Highlights

- Geographic tongue affects more females compared to male counterparts.
- Geographic tongue predominantly affects individuals below 30 years of age.
- In most cases, the geographic tongue is asymptomatic.
- A mixture of aspirin and ethanol is effective in managing symptomatic geographic tongue.

Geographic tongue, also known as benign migratory glossitis is a chronic, immune-mediated inflammatory disorder^1,2^. It is characterized by a decrease in the number of papillae (particularly the filiform papillae) in the dorsum and lateral borders of the tongue due to lymphocytic response^3,4^. Clinically, it appears as diffuse red round patches with white distinct borders giving the tongue a map-like appearance^1,2^.

Geographic tongue is more prevalent in individuals under 30-year-old, with a slight female predilection^3,5,6^. In most cases, the geographic tongue is asymptomatic. However, some of the common symptoms associated with it are a burning sensation, taste impairment, and tongue pain^1,3,7^. Though its etiology is unknown, some etiological factors attributed to it include emotional stress, genetic factors, vitamin deficiency, allergy, and immune disorders^1,3,5,7^. Others are bacterial or fungal infection, psoriasis, fissured tongue, hormonal disturbances, and diabetes mellitus^1,3,5,7^.

There is still no definitive cure for geographic tongue^1,6^. The treatment is mainly for symptomatic cases. Treatment options include topical steroids, retinoic acid, cyclosporine, antihistamine, tacrolimus, and immune system regulators. Purani and Purani^8^ and Ishibashi *et al.* ^9^ the success of topical tacrolimus on the management of geographic tongue. In another study, Aung-din *et al.* ^2^ reported a series of geographical tongue cases managed by the use of tacrolimus 2 mg/l swish-and-spit solution. In another study, Vahedi and colleagues^10^ used oral zinc sulfate in treating patients with geographic tongue. Despite several treatment options, they are neither specific nor curative^1,5^ and are limited, costly, and lack sufficient outcome data^2^.

It has been reported that the geographic tongue has histopathological similarity to psoriasis^11^, which has been shown to respond to salicylic acid (which is the principal metabolite of aspirin)^12,13^. Therefore, this article aims to evaluate the effectiveness of an aspirin-ethanol combination in the management of geographic tongue. It explores this as another treatment option that can be used in the management of geographic tongue.

## Methods

This was a prospective study of patients diagnosed of medical records of patients diagnosed with geographic tongue between 2018 and 2021. The inclusion criteria were patients aged 15 years and above with symptomatic geographic tongue and who had been treated in the past without improvement. Moreover, a minimum of 1 month had elapsed from the last administration of prior medications.

**Figure 1:**
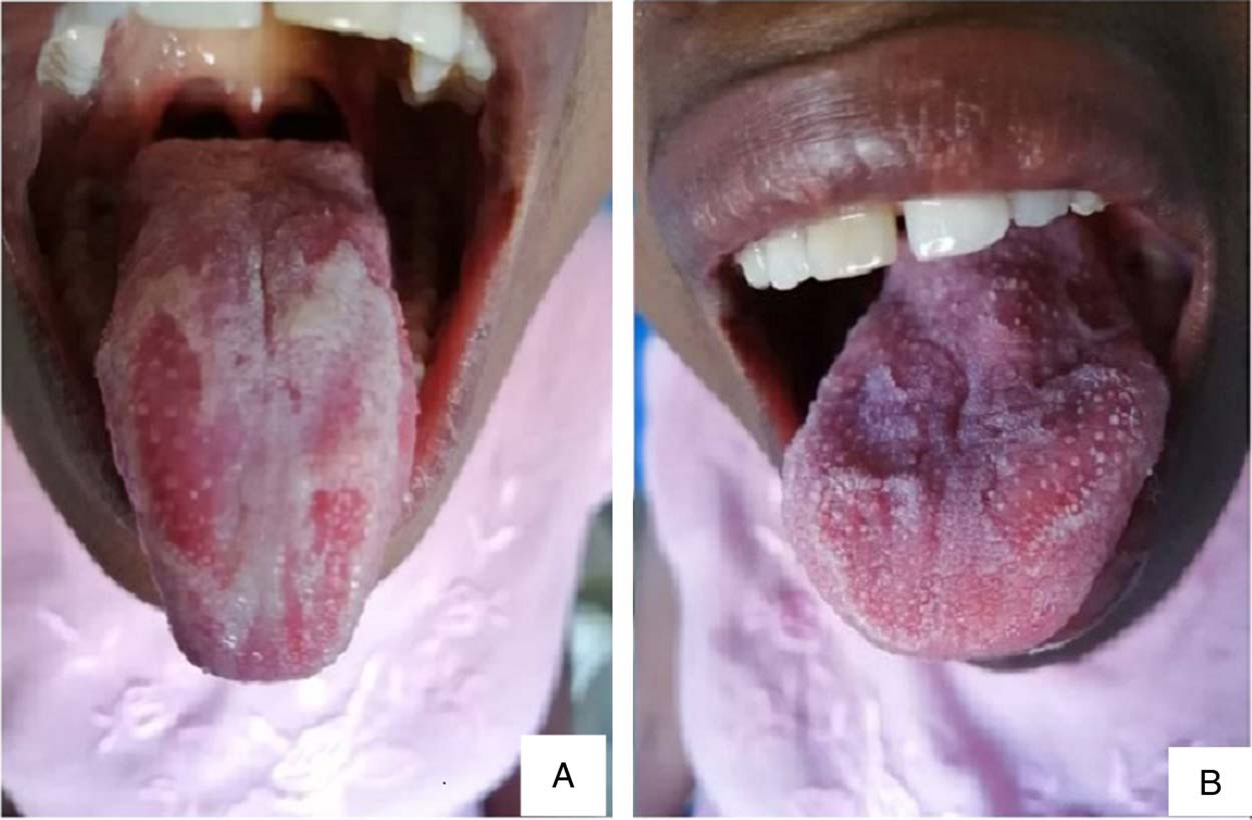
(A) Clinical appearance of geographic tongue in a patient before treatment. (B) Clinical appearance of the same patient with geographic tongue 5 min after treatment.

The treatment regimen involved dissolving 3 mg of aspirin (acetylsalicylic acid) into 1 ml of 70% v/v ethanol. Then a sterile gauze was soaked in the prepared mixture and topically applied with gentle pressure on the tongue surface for 2–3 min. The patients were instructed to leave their mouths open with their tongues protruding for about a minute (Fig. 1). The patients were thereafter instructed to self-cleanse the tongue at home by soaking a soft-bristled toothbrush in the mixture at least twice a day and to wait for about 30 min before ingesting anything after each session. This was to be done for a period of 5 to 7 days.

The intensity of oral discomfort and burning sensation was assessed using the Visual Analog Scale (VAS), which is a continuous scale comprised of a horizontal line of 10 cm in length, anchored by ‘no discomfort and burning sensation’ (score of 0) and ‘severe discomfort and burning sensation’ [score of 10 (10 cm scale)]. The respondent was asked to place a line perpendicular to the VAS line at the point that represents their discomfort and burning sensation intensity. The VAS scale was graduated into 10 intervals for ease of understanding for the patients. Before administering the questionnaire, patients were given an explanation and demonstration of how to score the VAS scale. The intensity of discomfort and burning sensation was assessed before treatment, one week after treatment, and during the third month follow-up. The treatment was considered successful if the patient scored the intensity of discomfort and burning sensation below 3 during the third month’s follow-up visit. Following a complete response to treatment, there was a yearly follow-up for possible recurrence.

The data obtained from this study were coded and analyzed using Statistical Package for Social Sciences software (SPSS) for Windows (version 26 IBM Corp), and were presented using frequencies, means, and percentages in the form of a table. Ethical clearance was sought from the Institutional Review Board (DA.282/298/01L/74), and permission to conduct the study was obtained from the appropriate authorities of the department of oral and maxillofacial surgery. The study fulfills the strengthening the reporting of cohort, cross-sectional and case-control studies in surgery (STROCSS) criteria for cross-sectional studies^14^.

## Results

The records of 23 patients who met the inclusion criteria were analyzed. There were more females (19, 82.6%), and male to female ratio of 1: 4.7. The age range of patients was 15–43 years (mean age of 23.43±1.50 years). Oral discomfort and burning sensation of the tongue were the symptoms reported by all (*N*=23, 100%) patients. Other symptoms included loss of taste (*N*=5, 21.7%) and pain (*N*=2, 8.7%)[Table 1].

**Table 1:** Patients’ characteristics, prior treatment, and treatment outcomes for geographic tongue

Case No	Sex	Age (years)	Accompanied symptoms	Duration of symptoms (months)	Prior treatment	Improvement on follow-up (minimum 3 months)
1.	F	16	Oral discomfort and burning sensations of the tongue, loss of taste.	4	Mouthwashes, antifungals.	Yes
2.	F	18	Oral discomfort and burning sensations of the tongue.	7	Mouthwashes, antifungals, analgesics.	Yes
3.	M	17	Oral discomfort and burning sensations of the tongue.	13	Mouthwashes, multivitamins, antifungals.	Yes
4.	F	25	Oral discomfort and burning sensations of the tongue.	5	Mouthwashes, antifungals.	Yes
5.	F	37	Oral discomfort and burning sensations of the tongue.	4	Mouthwashes, antifungals.	Yes
6.	F	28	Oral discomfort and burning sensations of the tongue.	10	Mouthwashes, multivitamins, antifungals.	Yes
7.	F	24	Oral discomfort and burning sensations of the tongue.	3	Mouthwashes, steroids, antifungals.	No
8.	M	19	Oral discomfort and burning sensations of the tongue.	6	Mouthwashes, antifungals.	Yes
9.	F	43	Oral discomfort and burning sensations of the tongue, loss of taste.	7	Mouthwashes, antifungals, analgesics.	Yes
10.	F	32	Oral discomfort and burning sensations of the tongue, pain.	8	Mouthwashes, analgesics.	Yes
11.	F	26	Oral discomfort and burning sensations of the tongue, loss of taste.	4	Antifungals and multivitamins.	Yes
12.	F	29	Oral discomfort and burning sensations of the tongue.	3	Multivitamins, mouthwashes, antifungals.	Yes
13.	M	24	Oral discomfort and burning sensations of the tongue.	6	Mouthwashes, multivitamins.	Yes
14.	F	23	Oral discomfort and burning sensation, pain.	3	Mouthwashes, antifungals.	Yes
15.	F	25	Oral discomfort and burning sensations of the tongue.	2	Mouthwashes.	Yes
16.	F	27	Oral discomfort and burning sensations of the tongue.	3	Mouthwashes, steroids.	Yes
17.	F	25	Oral discomfort and burning sensation, pain.	7	Mouthwashes, analgesics.	Yes
18.	F	18	Oral discomfort and burning sensations of the tongue.	2	Mouthwashes, antifungals.	Yes
19.	F	17	Oral discomfort and burning sensations of the tongue.	8	Mouthwashes, antifungals.	Yes
20.	F	16	Oral discomfort and burning sensations of the tongue.	2	Analgesics, multivitamins.	No
21.	M	18	Oral discomfort and burning sensations of the tongue.	4	Mouthwashes, analgesics, antifungals.	Yes
22.	F	15	Oral discomfort and burning sensations of the tongue.	2	Antifungals.	Yes
23.	F	17	Oral discomfort and burning sensations of the tongue.	3	Mouthwashes, analgesic.	Yes

The duration of symptoms ranged from 2 months to 13 months, with a mean of 5.17±0.59 months. Before the current treatment modality, all patients had undergone one or a combination of several therapies which included mouthwashes (*N*=20, 87%), antifungals (*N*=13, 56.5%), analgesics (*N*=8, 34.8%), multivitamins (*N*=7, 30.4%), and steroids (*N*=3, 13%)[Table 1].

The oral discomfort and burning sensation intensity score before treatment ranged between 6 and 9 with a median score of 8. At 1-week follow-up, oral discomfort and burning sensation intensity score ranged between 1 and 8 (median 3), and at 3 months follow-up the range was 0–7 (median 2). Only 2 (8.7%) patients reported poor response at follow-up (3 months after therapy)[Table 1].

The only adverse event most commonly reported by the patients was a raw and painful tongue during the application of the medication. However, the pain subsided 1–2 min after the application of the mixture.

## Discussion

Geographic tongue is among the inflammatory diseases of the oral mucosa that commonly affect the dorsal surface and lateral borders of the tongue^9,10^. This condition has also been referred to as erythema migrans, superficial migratory glossitis, lingual dystrophy, marginal exfoliative glossitis, and glossitis areata migrans^15^. Similar to the findings of this study, it has been documented in several works of literature that geographic tongue predominantly affects individuals below 30 years of age with a slight female predilection^3,5–7^. The role of hormones in the intensification of the condition has been proposed to explain females’ preponderance^5^.

Usually, geographic tongue is an asymptomatic condition that requires no treatment, however, reassurance to the patient and periodic follow-ups are recommended^16^. In symptomatic cases, several treatment options are opted for including medications like acetaminophen, anti-inflammatory drugs, antihistamines, topical steroids, cyclosporine, multivitamins, and immune system regulators^1,5,6,16^. However, to date, there is still no definitive cure for geographic tongue^1,6^.

**Figure 2:**
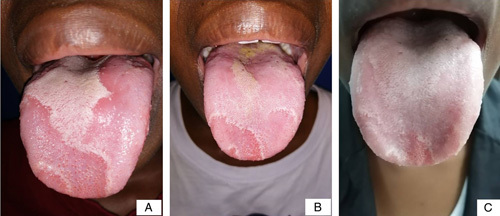
(A) Clinical appearance of geographic tongue in a patient before treatment. (B) Clinical appearance of the same patient with geographic tongue 3 days after treatment. (C) Clinical appearance of the same patient with geographic tongue 3 months post-therapy.

We use various therapies for managing symptomatic geographic tongue at our center. For the cases, which do not respond to the already documented therapies, a mixture of acetylsalicylic acid (aspirin) and ethanol was tried. The outcome of using the aspirin-ethanol mixture has proven to be effective, considering above 90% of the patients reported to be asymptomatic at 3 months follow-up (Fig. 2). The success of the proposed treatment modality in this study was almost similar to the results of Vahedi *et al.* ^10^ who used oral zinc sulfate but higher than the findings of Aung-din *et al.* ^2^ reported a series of geographical tongue cases managed by the use of tacrolimus 2 mg/l swish-and-spit solution.

Though long-term use of alcohol is associated with glossitis^17^, and there is a report showing daily use of alcohol-containing mouth rinses induced distortion of the dorsal surface of the tongue^18^. In our case, we used the treatment for a very short time of 5–7 days, which may not cause any significant changes to the tongue.

The rationale for using the aspirin-ethanol mixture is based on reports that geographic tongue has histopathological similarity to psoriasis^11^. It is also known that salicylic acid (which is the principal metabolite of aspirin) has been used effectively in the management of various dermatological conditions^19,20^ including psoriasis^12,13^.

Aspirin is readily absorbed in the upper gastrointestinal tract and metabolized in the liver^20^. Salicylic acid has been shown to reduce the intercellular cohesiveness of the horny cells and tends to reduce the pH of the stratum corneum, thereby increasing hydration and softening, and thereby reducing the amount of cellular shedding^13^. Moreover, aspirin acts by irreversibly inhibiting both cyclooxygenase enzymes leading to a decreased production of prostaglandins hence decreasing inflammation^20^.

On the other hand, ethanol (alcohol) is used as a carrying agent for aspirin because it increases oral mucosal permeability by destroying the lipid composition that forms a protective layer of the oral mucosa and disrupting the normal order of epithelial lipid molecules, resulting in a gap between epithelial cells^21^.

The only setback of this treatment option is that it causes stinging at the site of application, though it is of few seconds, yet still, this option is worth using, especially in settings of developing countries where due to low socioeconomic status, accessibility of other types of medications for the geographic tongue is either limited or costly.

## Conclusion

To our knowledge, this is the first report regarding the use of an aspirin-ethanol mixture for treating symptomatic geographic tongue. A short-term application of the combination has shown to be effective. We recommend a multicentric clinical trial to study the efficacy and safety of a mixture of aspirin and ethanol in managing symptomatic geographic tongue.

## Ethical approval

Ethical clearance was sought from the Institutional Review Board (DA.282/298/01L/74).

## Consent

NA.

## Sources of funding

None.

## Authors contribution

K.S.S.: conceived the idea, guided the whole process of data collection, data analysis, writing, and editing of the first draft, and approved the final draft; S.S.O.: broadened the conceived idea, guided the whole process of data collection and writing and editing of the first draft and approved the final draft; J.R.M. and W.A.O.: broadened the conceived idea, writing, and editing of the first draft and approved the final draft; B.L.: guided the whole process of data collection and reading and approved the final draft.

## Conflicts of interest disclosure

None.

## Guarantor

Karpal Singh Sohal.

## Provenance and peer review

Not commissioned, externally peer-reviewed.

## References

[1] Najafi S Gholizadeh N Akhavan Rezayat E . Treatment of symptomatic geographic tongue with triamcinolone acetonide alone and in combination with retinoic acid: a randomized clinical trial. J Dent (Tehran) 2016;13:23–28. 27536325 PMC4983562

[2] Aung-Din D Heath M Wechter T . Effectiveness of the tacrolimus swish-and-spit treatment regimen in patients with geographic tongue. JAMA Dermatol 2018;154:1481. 30419108 10.1001/jamadermatol.2018.3806 PMC6583324

[3] Picciani BLS Santos LR Teixeira-Souza T . Geographic tongue severity index: a new and clinical scoring system. Oral Surg Oral Med Oral Pathol Oral Radiol 2020;129:330–338. 31974034 10.1016/j.oooo.2019.12.007

[4] González-Álvarez L García-Pola MJ García-Martín JM . Geographic tongue: predisposing factors, diagnosis and treatment. A systematic review, Rev. Clínica Española (English Ed 2018;218:481–488. 10.1016/j.rce.2018.05.006 29903400

[5] Honarmand M Farhad Mollashahi L Shirzaiy M . Geographic tongue and associated risk factors among iranian dental patients. Iran J Public Health 2013;42:215–219. 23515238 PMC3595651

[6] Shulman J Carpenter W . Prevalence and risk factors associated with geographic tongue among US adults. Oral Dis 2006;12:381–386. 16792723 10.1111/j.1601-0825.2005.01208.x

[7] Oyetola EO Oluwande A Agho ET . Geographic tongue: pattern of presentation in a South Western Nigerian Teaching Hospital., Ann. Ibadan. Postgrad Med 2018;16:131–135. PMC6580408 31217770

[8] Purani JM Purani HJ . Treatment of geographic tongue with topical tacrolimus. Case Reports 2014;2014:bcr2013201268–bcr2013201268. 10.1136/bcr-2013-201268 PMC4127763 25085945

[9] Ishibashi M Tojo G Watanabe M . Geographic tongue treated with topical tacrolimus. J Dermatol Case Rep 2011;4:57–59. 10.3315/jdcr.2010.1058 PMC3157822 21886753

[10] Vahedi M Abdolsamadi HR Mortazavi H . Evaluation of the therapeutic effects of zinc sulfate in patients with geographic tongue. Avicenna J Dent Res 2009;1:11–14.

[11] Picciani BLS Teixeira-Souza T de HF . Geographic tongue and psoriasis: clinical, histopathological, immunohistochemical and genetic correlation – a literature review. An Bras Dermatol 2016;91:410–421. 27579734 10.1590/abd1806-4841.20164288 PMC4999097

[12] Gooderham M Blakely K . Management of scalp psoriasis: current perspectives. Psoriasis Targets Ther 2016;6:33. 10.2147/PTT.S85330 PMC5683126 29387592

[13] Lebwohl M . The role of salicylic acid in the treatment of psoriasis. Int J Dermatol 1999;38:16–24. 10065604 10.1046/j.1365-4362.1999.00500.x

[14] Mathew G Agha R Albrecht J . STROCSS 2021: strengthening the reporting of cohort, cross-sectional and case-control studies in surgery. Int J Surg 2021;96:106165. 34774726 10.1016/j.ijsu.2021.106165

[15] Assimakopoulos D Patrikakos G Fotika C . Benign migratory glossitis or geographic tongue: an enigmatic oral lesion. Am J Med 2002;113:751–755. 12517366 10.1016/s0002-9343(02)01379-7

[16] Nandini DB . Paediatric geographic tongue: a case report, review and recent updates. J Clin Diagnostic Res 2016;10:ZE05–ZE09. 10.7860/JCDR/2016/16452.7191 PMC4800664 27042597

[17] Ivoš A Matošić A Gradiški IP . The effects of alcohol on oral health, a review. Arch Psychiatry Res 2019;55:61–70.

[18] Hamzawy MA Shehata N El Zainy M . Impact of daily using alcohol-containing mouthwashes on tongue, cementum, and enamel surface; in vitro and in vivo studies. Clin Exp Pharmacol 2017;07:1–7.

[19] Arif T . Salicylic acid as a peeling agent: a comprehensive review. Clin Cosmet Investig Dermatol 2015;8:455. 10.2147/CCID.S84765 PMC4554394 26347269

[20] Bubna AK . Aspirin in dermatology : Revisited. Indian Dermatol Online J 2015;6:428–435. 26753146 10.4103/2229-5178.169731 PMC4693360

[21] Feng L Wang L . Effects of alcohol on the morphological and structural changes in oral mucosa. Pak J Med Sci 2013;29:1046–1049. 24353685 10.12669/pjms.294.3696 PMC3817782

